# Molecular Network for Regulation of Ovule Number in Plants

**DOI:** 10.3390/ijms222312965

**Published:** 2021-11-30

**Authors:** Muslim Qadir, Xinfa Wang, Syed Rehmat Ullah Shah, Xue-Rong Zhou, Jiaqin Shi, Hanzhong Wang

**Affiliations:** 1 Key Laboratory of Biology and Genetic Improvement of Oil Crops, Ministry of Agriculture, Oil Crops Research Institute of the Chines Academy of Agricultural Sciences, Wuhan 430062, China; msmirwani22@gmail.com (M.Q.); wangxinfa@caas.cn (X.W.); 2 Department of Plant Breeding and Genetics, Faculty of Agriculture, Lasbela University of Agriculture Water and Marine Sciences (LUAWMS), Lasbela 74200, Pakistan; srushah@gmail.com; 3 Department of Soil and Environment, Swedish University of Agricultural Sciences, P.O. Box 7080, SE-75007 Uppsala, Sweden; 4 Commonwealth Scientific Industrial Research Organization (CSIRO) Agriculture Food, Canberra, ACT 2601, Australia; xue-rong.zhou@csiro.au

**Keywords:** ovule number genes, molecular network, auxin, cytokinins, brassinosteroids, gibberellin, transcription factors, micro-RNA

## Abstract

In seed-bearing plants, the ovule (“small egg”) is the organ within the gynoecium that develops into a seed after fertilization. The gynoecium located in the inner compartment of the flower turns into a fruit. The number of ovules in the ovary determines the upper limit or the potential of seed number per fruit in plants, greatly affecting the final seed yield. Ovule number is an important adaptive characteristic for plant evolution and an agronomic trait for crop improvement. Therefore, understanding the mechanism and pathways of ovule number regulation becomes a significant research aspect in plant science. This review summarizes the ovule number regulators and their regulatory mechanisms and pathways. Specially, an integrated molecular network for ovule number regulation is constructed, in which phytohormones played a central role, followed by transcription factors, enzymes, other protein and micro-RNA. Of them, AUX, BR and CK are positive regulator of ovule number, whereas GA acts negatively on it. Interestingly, many ovule number regulators have conserved functions across several plant taxa, which should be the targets of genetic improvement via breeding or gene editing. Many ovule number regulators identified to date are involved in the diverse biological process, such as ovule primordia formation, ovule initiation, patterning, and morphogenesis. The relations between ovule number and related characteristics/traits especially of gynoecium/fruit size, ovule fertility, and final seed number, as well as upcoming research questions, are also discussed. In summary, this review provides a general overview of the present finding in ovule number regulation, which represents a more comprehensive and in-depth cognition on it.

## 1. Introduction

**Figure 1:**
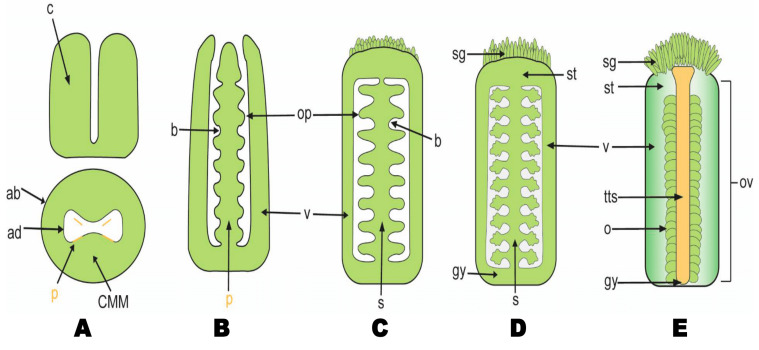
Model diagram elaborating ovule formation in Arabidopsis. The instance specifies ovule formation and developmental stages. (**A**) CMM formation, (**B**) ovule identity, (**C**) ovule initiation, (**D**) integument development, and (**E**) gynoecium development. Abbreviations: ab, abaxial; ad, adaxial; b, boundary; CMM, carpel margin meristem; c, carpel; gy, gynoecium; o, ovule; op, ovule primordium; ov, ovary; p, placenta; s, septum; sg, stigma; st, style; tt, transmitting tract; v, valve. The region of the CMM where the placenta is formed is indicated with orange lines. The ovule initiation and gynoecium developmental process are according to [3,6].

The reproductive organs formation and their meiocytes take place late during plant development, in contrast to animals where primordial germ cell development occurs during embryonic development [1,2]. In flowering plants, the ovule is located inside the ovary of the gynoecium, which ultimately becomes the fruit upon pollination and ovule fertilization. The ovule has simple yet highly differentiated architecture (Figure 1). It consists of three regions/sectors: the funiculus, bridging the ovule to the placenta; the chalaza, forming the integument(s); and the nucellus covered by the integuments. The nucellus represents the most important compartment of the ovule where the megaspore mother cell differentiation into the embryo sac occurs [3,4]. After the egg cell in the embryo sac has been fertilized, the ovule develops into a seed [5]. In some plants, several egg cells exist in the ovary.

Ovule initiation and developmental processes have been extensively examined at the morphological, genetic, and molecular levels in Arabidopsis and other plant species [7,8,9,10,11,12]. These studies show the formation of a completely developed set of ovules through several fundamental processes, including three main stages. These stages are primordia initiation and extension from the carpel margin meristem (CMM) at the placental tissue within the developing carpel, sporogenesis, giving rise to the large subepidermal megaspore mother cell at the tip of the primordium, and meiosis, resulting in a tetrad of haploid megaspores. Only one of the megaspores is functional and undergoes differentiation for the embryo sac or gametophyte [5,11]. The embryo sac harbors the reproductive cells due to three rounds of mitotic divisions accompanied by cellularization. In Arabidopsis, integuments develop asymmetrically leading to the anatropy (curvature characteristic) of the mature ovule [13]. All of these developmental processes are controlled by the complex interaction between phytohormones and signaling networks [14,15,16,17,18]. 

The ovule number and fertility are directly reflecting the developmental consequence of ovules in a quality and quantity manner, which are mainly determined by the ovule initiation and subsequent megasporogenesis/megagametogenesis processes, respectively. From an evolutionary point of view, selection favors more ovules and potentially more seeds due to its higher reproduction efficiency and fitness [19]. From an agronomical point of view, the ovule number per ovary (ONPO) sets the upper limit of seed number per fruit (SNPF). An optimal balance/trade-off between the ovule/seed number and seed size is needed to achieve the required breeding objectives [15]. As a result, the ovule number is an important genetic trait for plant evolution and crop improvement [20,21]. Understanding the mechanism of ovule number regulation has emerged as a significant research aspect in plant science.

The ovule number per flower varies over several orders of magnitude among the different taxa of angiosperms [19,20,21]. A wide variation of ovule numbers has also been shown among the different accessions of the same species [22,23,24,25]. In addition, ONPO also varies according to the flower position and inflorescence, linked to the distribution of assimilates towards the sink [26,27,28]. Both genetic and environmental factors control the natural variation of ONPO. As a typical quantitative trait, ONPO has been subjected to genetic dissection using linkage and/or association mapping in recent years [21,22,29,30]. Although dozens of QTLs for ovule number have been identified in several plant species, only one (*NERD1*) has been cloned [22]. *NERD1* encodes an integral membrane protein that positively controls flower number, ovule number, and productivity in Arabidopsis. However, analyses of mutants defective in ovule development have identified dozens of genes affecting ONPO, mainly from Arabidopsis, but also in petunia, rice, tomato, and rapeseed [31]. The summary of these ovule number genes, emphasizing their regulatory pathways and molecular mechanisms, will provide a comprehensive and in-depth understanding of ovule number regulation in plants.

The objectives of this review are to (1) comprehensively collect ovule number regulators known to date; (2) summarize their regulatory pathways and molecular mechanisms; (3) construct an integrated molecular network for them; (4) explore their functional conservation across different species or pleiotropy on other characteristics/traits. This review will provide novel, systematic, and further insights into the genetic regulation of ovule numbers at the molecular level in higher plants. We also give some creative suggestions and practical targets toward the molecular improvement of ovule numbers with technologies such as gene editing, which will be very useful for plant breeders. 

## 2. Molecular Network of Ovule Number Regulation 

**Figure 2:**
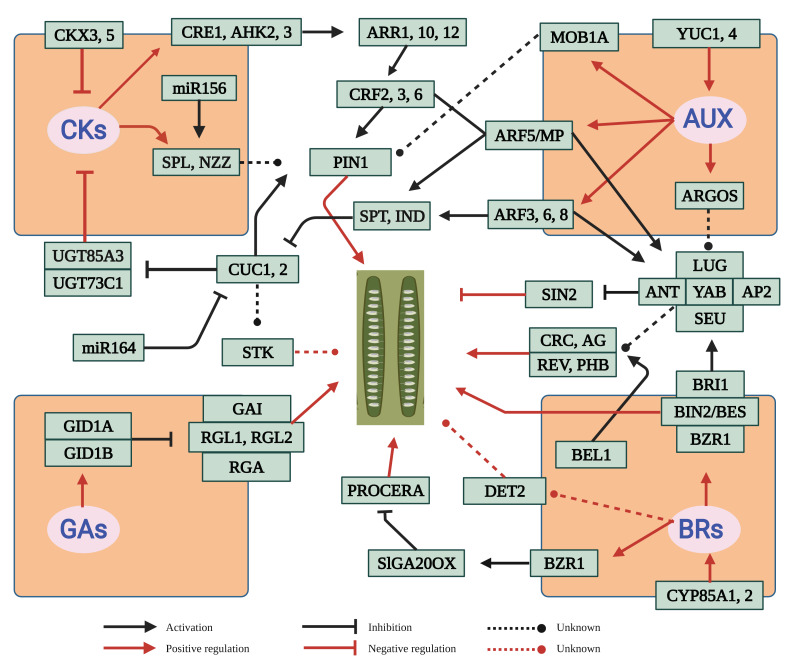
An integrated gene network for the regulation of ovule number. Four types of phytohormones (AUX, BRs, CKs and GAs) and other regulators are shown in blue and black color, respectively. The black and red arrows show the relationship between the up-/down-stream genes and between genes and phenotype, respectively.

Much research on ovule development has shown that ovule number is determined at early stages of floral development, such as CMM and placenta formation, ovule identity, primordium initiation, pattern formation [3,31], etc. Several genes that can affect ovule number have been reported, mainly from the characterization of mutants in *Arabidopsis* *thaliana* (Table 1). Functional characterization of “ovule number controlling genes” shows that a significant number play a role in biosynthesis and signaling pathways of several types of phytohormones, mainly as auxins (AUX), cytokinins (CK), brassinosteroids (BR), and gibberellins (GA). Receptor proteins perceive these hormones, subsequently initiate intracellular signal transduction and transcription factors (TFs), and finally activate the downstream hormonal response. Other proteins and small RNAs also participate in the regulation of ovule number through interplaying with phytohormones and transcription factors (Figure 2; Table S1).

### 2.1. Auxins—A Positive Regulator of Ovule Number

Auxins are the main phytohormones involved in plant cell division and expansion to drive diverse processes of plant growth and development (e.g., embryogenesis, growth patterning, and tissue differentiation) in a concentration- and cell-type-dependent manner [75]. Many genes involved in the auxin pathway (such as: biosynthesis, homeostasis, transport, signal transduction, and downstream response) are shown to have an effect on ovule number [31]. 

The biosynthesis of most auxins in plants depends on the YUCCA (YUC) family of flavin-binding monooxygenase. YUC proteins are responsible for the conversion of indole-3-pyruvate acid (IPyA) to form indole-3-acetic acid (IAA) in a rate-limiting manner [75]. The Arabidopsis *yuc1yuc4* double mutant showed multiple growths and developmental defects, including the reduction or absence of placental tissue and ovules [40,43]. 

In response to auxin, the *AUXIN RESPONSE FACTOR 5* (*ARF5*)*/MONOPTEROS* (*MP*) is an essential integrator of auxin signaling in Arabidopsis by activating gene transcription in cells. Multiple transcription factors, including *CUP-SHAPED COTYLEDON1* *(CUC1)*, *CUC2*, *AINTEGUMENTA* (*ANT*), *CYTOKININ RESPONSE FACTOR 2* (*CRF2*), and *DORNROSCHEN* (*DRN*), are directly activated and controlled by the ARF5 gene [43,76,77]. These genes redundantly regulate the expression of *PINFORMED1* (*PIN1)*, which is one of the eight transmembrane auxin transporters in Arabidopsis and is required for auxin gradients and ovule primordium formation [3,78,79]. The *PIN1* efflux transporter is involved in polar auxin transport, which is needed to initiate a local maximum concentration of auxin in the placenta, where the specialization of founder cells of the ovule primordia occurs [43,78,79]. A partial loss-of-function mutation mp-S319 allele results in a reduced number of organs, such as a few flowers with missing carpel margin tissue, placenta, and ovules [76,80]. In addition, *AUXIN RESPONSE FACTOR 3* (*ARF3*) / *ETTIN* (*ETT*) is also activated by auxin and down-regulates the expression of *SPATULA* (*SPT*), a bHLH transcription factor [34,35,48] that represses the expression of *CUC1* and *CUC2* [61]. The transmission tract, style/stigma formation, and ovule number are regulated through ARF8, *ARF6*, and *ARF3/ETT.* However, these three genes are also involved separately in the control of carpel development. The lineage leading to *ETT* is seed plant-specific, while that leading to *ARF6* and *ARF8* has been reported in ferns [18,81]. The *ett* mutations have a pleiotropic impact on Arabidopsis development, containing elongated style and gynophore, and a shorter ovary with fewer ovules [18,34,82]. Mutations in *SPT* cause a minor loss in ovule number through the formation of a split-carpel phenotype at the top of the gynoecium [61,83].

*SEUSS(SEU)* belongs to a transcriptional co-regulators family and is needed for an effective response to auxin signaling. The combined loss of *SEU* and *ANT* activity resulted in the complete loss of ovule formation [7,84,85]. The *seu ant* quadruple mutants displayed a considerable reduction in the number of initiated primordia ovules compared to the single mutants and wild type [41,86]. *LEUNIG* (*LUG*) is a transcriptional co-repressor involved in gynoecium marginal tissue development. Its combinational mutation with *ant* fails to develop placentas and ovules [63]. SEU and LUG are also involved in the development of the central domain of the gynoecium [7,63]. The transcription factor PERIANTHIA (PAN) plays a role in developing the gynoecium medial domain and the formation of ovule primordia [33]. Mutations in the PAN gene cause a significant increase in the number of ovules in the *ant seu* double mutants. *PAN* is a bZIP transcription factor expressed in the gynoecium medial ridge and the placenta, where it stimulates the formation of ovules [31,33]. This PAN function was disclosed in *pan* *ant* and *seu pan* double mutants that showed a reduced number of ovules [33]. 

*CUC1* and *CUC2* are the major contributors to ovule initiation, and their double mutant showed reduced ovule number [45,87]. In addition, *CUC1* and *CUC2* are regulated post-transcriptionally by *miR164*, and the over-expression of *miR164* reduces ovule number greatly [88]. More importantly, both *CUC1* and *CUC2* are involved in regulating the expression and localization of *PIN1*. The *pin1* mutant shows reduced auxin transport activity and multiple growth and developmental defects, including reduced ovule number [32,89]. 

### 2.2. Cytokinins—A Positive Regulator of Ovule Number

Cytokinins (CKs) are a major class of phytohormones with diverse molecular structures, essential for plant physiological and developmental processes [90,91,92]. Many of the genes that are involved in CK pathway (signal transduction, biosynthesis, transport, homeostasis, and degradation) plays an important role in determining ovule number [49,93,94]. 

The Cytokinin Oxidase/Dehydrogenase (CKX) gene family, which consists of seven members *CKX1* to *CKX7* in *Arabidopsis*, encodes for enzymes that catalyze the metabolic degradation of CKs [46,93]. Theckx3 and ckx5 double mutant in Arabidopsis develops twice as many ovules as the wild type [31,47]. These mutants show accumulation of CK content and higher inflorescence meristematic activity, resulting in a higher number and larger flowers/siliques with more ovules/seeds and higher seed yield [47]. 

Cytokinin Response Factors (CRFs) are a subgroup of AP2/ERF transcription factor genes in Arabidopsis. CRFs genes are reported to control a significant proportion of the CK transcriptional response that overlaps functionally with the B-type ARR-mediated response [49,95]. *CRF2*, *CRF3*, and *CRF6* redundantly stimulate the expression of *PIN1*, supporting the critical role of CRF factors as mediators of AUX/CKs crosstalk controlling plant organogenesis [49]. The triple mutant of *crf2*, *3*, *6* shows a reduced number of ovule and pistil length, and a shorter placenta [49,94]. However, the placenta size was not enough to explain the 30% decrease in ovule number as ovule density was lower in *crf2/3/6* compared to the wild-type [31,49]. 

Arabidopsis Response Regulators (ARRs) A and B are involved in CK signaling. The B-type ARRs are induced by phosphorylation of the Asp receptor in their binding domain by AHPs, which are important to initiate the transcriptional response. The ARR B-type triple mutant *arr1*, *10*, *12* exhibits reproductive defects, containing shorter gynoecium and flower length, reduced replum width, and fewer ovules [31,48]. In addition, the ovule number reduction phenotype in arr1 and arr12 mutants can be rescued by CK exogenous treatment [43]. This evidence suggests the existence of a cross-talk of both AUX and CK in the regulation of ovule development. The histidine kinases are CK receptors, encoded by eight canonical members *AHK1-5*, *ETR1*, *ERS1*, and *CKI1* [96,97]. The triple mutant *ahk2*, *3*, *4* shows defects in the formation of female gametophyte (FG) and growth arrest at stage FG1-FG2 [98], resulting in a substantial reduction in the number of ovules [22,42,99]. 

Cucinotta et al. [1] found that mutation of *CUC1* and *CUC2* influences CK homeostasis. They evaluated the CK content in the double mutant and conducted a transcriptomic analysis to figure out the disrupted genes that function in CK homeostasis and influence ovule number control. The *UGT85A3* and *UGT73C1*, encoding *udp-glucosyl transferase* that is able to catalyze CK inactivation by O-glucosylation, are up-regulated without *CUC1* and CUC2 [1]. A moderate increase in ovule and seed number was observed in *ugt85a3* mutant lines, compared to wild type [1,31,43]. 

### 2.3. Brassinosteroids (BRs)—A Positive Regulator of Ovule Number

Brassinosteroids are plant-specific steroidal hormones categorized by their polyhydroxylated sterol structure and were initially identified from rapeseed crop pollen [100,101]. BRs control numerous biological functions, including seed germination, cell elongation, root growth, photomorphogenesis, reproduction, and multiple biotic and abiotic stresses [101]. Many genes involved in the synthesis and signal transduction of BRs have been shown to play a role in regulating ovule number [100,102,103]. 

The synthesis of the two main active BRs, castasterone (CS) and brassinolide (BL), in Arabidopsis requires the activity of *CYP85A2*, a rate-limiting factor for the conversion of 6-deoxyCS to CS and CS to BL [104]. Darker green and rounder leaf, shorter plant height and stamens with reduced male fertility, and decreased ovule number were observed in the single mutant of the *cyp85A2* gene [41]. *DEETIOLATED2* (*DET2*) gene encodes 3-Oxo-5-α-steroid 4-dehydrogenase, which is involved in the BL biosynthetic process in Arabidopsis. The loss-of-function *det2* mutant led to a BR-deficient phenotype with considerable growth and developmental defects, including shorter stature, tiny leaves and flowers, reduced male fertility, and lower ovule number per pistil [50]. *BRI1* (*Brassinosteroids-Insensitive 1*) encodes a plasma-localized leucine-rich repeat receptor kinase, which binds BR and activates transduction, resulting in phosphorylation of the kinase domain leading to *BR* responses. The *bri1-5* mutant showed a BR-insensitive phenotype with shorter and thinner siliques and few ovules per pistil [50,105].

*BIN2* (*BR-INSENSITIVE 2*) encodes a member of the ATSK family, which negatively regulates the BR signal by interacting and phosphorylation of many other transcription factors that act synergistically with or independently of *BZR1/BES1*. The *bin2* mutant also showed a BR-insensitive phenotype with shorter pistil and fewer ovules [50]. *BZR1* *(Brassinazole-Resistant 1)* is a positive regulator of the BR signaling pathway that mediates both downstream BR response and negative feedback regulation of BR bio-synthesis. The gain-of-function *bzr1-1D* mutant exhibited enhanced BR signal with longer and thicker siliques, more crowded seeds, and more ovules per pistil [50]. 

BRs affect ovule number and gynoecium size by modulating gene expression, like *HLL* and *ANT*, which exhibit similar effects on primordial ovule growth [12,14,62], and the *AP2* gene, which affects ovule number determination and developmental specificity of the floral organ. As expected, the loss of function *ant* mutation affects carpel margin meristem and placenta development, resulting in abnormal lateral organs, and reduced fertility and number of ovules [31,41,106]. Previous studies have demonstrated that BR affects ovule number via transcriptional control of primary ovule development genes *ANT*, *HLL*, and *AP2* [12,50]. BZR1 activity was correlated with the upregulation of *ANT* and *HUL* genes, showing that BR signaling positively influences ovule number [50,57]. 

Multiple *ap2* mutant alleles were introduced to generate double mutants with *sap*. In Arabidopsis, a recessive mutation *STERILE APETALA* (*SAP*) causes inflorescence and ovule development aberrations. Concerning the two inflorescence organs, the genes appear to act synergistically, since *sap/ap2-1* double mutants never produce second whorl organs. The pistil is normal morphologically, although its size is significantly reduced, resulting in lower ovule number [72]. The ovule number of *bzr1-1D* and *ap2-5* single and double mutants, as well as other molecular evidence, reveals that *BZR1* and *AP2* have antagonistic effects on ovule number regulation [50,51].

*SHORT INTEGUMENTS2 (SIN2)* encodes a mitochondrial DAR GTPase and is reported to function in mitochondrial ribosome assembly like HLL [51]. The mutation in *SIN2* resulted in fewer ovules; however, disruption of SIN2 function caused an abnormal division of cells in the placenta [3,51,107]. 

### 2.4. Gibberellins (GA)—A Negative Regulator of Ovule Number

Gibberellins regulate major aspects of plant growth and developmental processes, including seedling and vegetative growth to flower maturity [108]. GAs are endogenous plant growth regulators, containing tetracyclic, diterpenoid substances, which were initially studied in 1950s [109]. GAs are involved in ovule primordia formation, thus affecting ovule number and development in Arabidopsis and tomato [27]. DELLA are regulatory proteins with a key role in GA signaling, acting as negative regulators, DELLA proteins coded by five genes (*GAI*, *RGA*, and three *RGA*-like, *RGL1*, *RGL2*, and *RGL3)* in Arabidopsis and *PROCERA* in tomato, which represses GA responses [14,53,110]. 

GAs and DELLA activity has been shown to be involved in ovule primordia initiation [27]. DELLA proteins have been discovered to be novel ovule number regulators in Arabidopsis, tomato, and rapeseed crops. The *global* mutant lacking the five DELLA of Arabidopsis (*gaiT6 rgaT2 rgl1-1 rgl2-1 rgl3-1*) produces fewer ovules. The quadruple (gaiT7 rgaT2 rgl1-1 rgl2-1) and triple (3xdella, gaiT6, rgaT2 rgl2-1) mutants show a comparable reduction in ovule initiation, suggesting that they have a significant role in ovule initiation. GA-treated plants were also shown to have a significant reduction in ovule number that largely recapitulated the null *della* mutants [27]. These gene were expressed in placental tissues and in outgrowing ovules [6,27,111]. The tomato *procera* mutant, lacking the activity of the single DELLA protein in the tomato genome, shows a reduced number of ovules. The ovule number is reduced to a similar extent in the *GA20ox* transgenic tomato line L4 [27], constitutively expressing *GA20* oxidase of GA biosynthesis and, therefore, accumulating high GA levels [27,55]. Loss-of-function mutations in DELLA genes (or GA treatments) cause a reduction in ovule number, whereas loss of function mutations in the GA receptors GID1A and GID1B promote ovule formation [27,77].

Two transcription factors, *REPRODUCTIVE MERISTEM 22 (REM22)* and *UNFERTI-LIZED EMBRYO SAC 16 (UNE16)*, were identified as putative DELLA targets by transcriptomic analysis of gai-1 and global della mutant stage 8–9 pistils [14,27,31]. REM22 is a B3 family transcription factor expressed in the placenta [56] and increased *REM22* expression in the *rem22-1* enhancer allele significantly increases ovule number [14,27,56,112]. UNE16 encodes homeodomain-like family transcription factors and its knock-down allele une16-1 produces fewer ovules. Because the expression of UNE16 is also regulated by BRs [91,92], it could be a hub for crosstalk between GAs and BRs during ovule initiation [26]. 

### 2.5. Other Ovule Number Regulators

Plant genomes contribute about 7% of their coding sequence to transcription factors, indicating the importance of transcriptional regulation [113,114]. In plants, animals, and fungi, MADS domain transcription factors are essential members of regulatory networks that regulate many developmental processes (Table 1) [115,116]. Systematic reverse genetic study of transcription factor families, such as the MADS domain proteins encoded by *STK* and *SHP1/2*, has revealed the critical ovule number genes. In Arabidopsis, the *STK*, *SHP1*, and *SHP2* genes have been demonstrated to have a significant impact on ovule number, identity, and carpel development [57,67,68,117]. The *AG* and *BEL1* genes are required for several aspects of ovule development, including specification of integument identity and, morphogenesis [1,118].The SEP1, SEP2, and SEP3 genes are closely linked to MADS box transcription factors, and are necessary for defining the identity of petals, stamens, carpels, and ovules, according to genetic analysis. [119]. In the *sep1*, *sep2*, *sep3* mutant, ovule number decreased, and a few other ovules lost their identity by transforming into carpel- and leaf-like structures, which provided genetic evidence for the significance of *SEP* proteins in the formation of an ovule identity-promoting complex [66].

The *ER* genes are homologous to receptor protein kinase and involved in the specification of organs originating from the shoot apical meristem synergistically with its paralogs *ERL1* and *ERL2* [69,120]. The reduction of ovule number reported in *er-105* appears to be an indirect result of the smaller fruit size and limited space availability [45]. When er-105 was combined with erl1-2 or erl2-1, the fruit length increased while the total ovule number was reduced, but interestingly, with an even higher ovule density. *EPFL2* (At4G37810) is a member of the EPF/EPFL (epidermal patterning factor/EPF-like) gene family, which encodes plant-specific secretory peptides. In the Ler background, the epfl2-1 mutation resulted in a modest fruit shortening but a considerable drop in ovule number and density when compared to Ler. [45,70]. The ASH1 protein family, which can methylate lysine residues on histone tails, helps Arabidopsis keep an effective transcriptional state during development. *ASHH2* has been characterized as a regulator of reproductive development through H3K36 trimethylation. Plants homozygous for *ashh2* null alleles exhibited an 80% reduction in ovule numbers relative to wild-type plants, and less than a quarter of the ovules developed into mature seeds [3,73]. 

*CRC* encodes plant-specific transcription factor YABBY family protein, which is involved in specifying abaxial cell fate in the carpel [121]. The *CRC* mutations showed numerous phenotypic variations compared to the wild type, including loss of determinacy and fusion, wider and shorter gynoecium/silique, decreased carpel and style height, and reduced ovule number [5,83,122]. 

The *miR156* is a highly conserved and expressed miRNA family in the plant that has been demonstrated to regulate multiple aspects in growth and development [16]. In a sbp8 mutant, overexpression of the miR156 resulted in fewer ovules, malformed septa, and a lack of transmitting tract tissue, as well as a shorter style. SBP and targets of miR156 from the SBP family can interact genetically with ettin/arf3, spt, and crc, implying that the miR156-SBP regulatory module regulates auxin homeostasis during gynoecium patterning [16]. 

## 3. Concluding Remarks and Future Research Directions 

**Figure 3:**
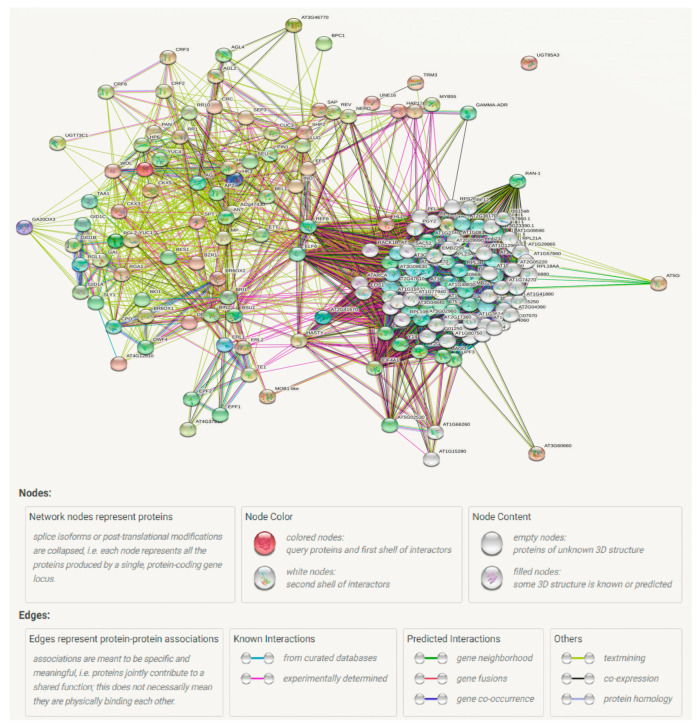
Protein interaction network constructed for ovule number genes. The figure shows different action types and effects, which are represented by different colors of lines and arrows between different genes/proteins. For example, the blue-colored lines/arrows show binding, green color represents activation while red color shows inhibition, black color represents reaction between different genes, and so on. The different types of arrows indicate the positive, negative and unspecified effects of genes.

Ovule number is not only a key characteristic related to plant adaptability/evolution [122], but also an important contributor to crop yield [15,31]. While there have been many studies reporting the individual genes associated with ovule number, no comprehensive review with further insights on its regulatory network is available. The extensive collection and further analysis of the genes affecting ovule number showed that the majority of them can be incorporated into an integrated molecular network (Figure 2 and Figure 3) that mainly involves four types of phytohormones including AUXs, CKs, BRs, and GAs [14]. This clearly demonstrates the central role of the four types of phytohormones in regulating ovule number. Further studies are then needed to investigate the relationship between this network and other ovule number genes. These genes can be targets for the molecular improvement of ovule number through over-expression or editing. In addition, AUXs, CKs, and BRs are the positive regulators of ovule number [14,16], whereas GAs act as a negative contributor [27]. Therefore, to obtain the most ovules, the four types of phytohormones must be coordinated. Another question that needs to be answered is whether other types of phytohormones also play a role in ovule number regulation. 

One of the major problems regarding the molecular improvement of ovule number is its relationship with other related traits, such as seed number, ovule fertility, and gynoecium/fruit size. Interestingly, most of the ovule number regulators collected in this review also affect gynoecium/fruit size, generally in the same direction (Supplementary Table S1), indicating a coordinated regulation between them. In addition, some previous studies that involve ovule number regulators have also investigated seed number. The results showed that these ovule number regulators also affect seed number, generally in the same direction, such as *AHK2*, *AHK3*, *AHK4/ CRE1*, *ASHH2*, *AP2*, *BIN2*, *BRI1*, *BZR1*, *DET2*, *HEMN1*, *RGL2*, *SPT*, *UGT85A3*, *UGT73C1*. These genes can be the optimal target for the genetic improvement of seed numbers. A key issue in using these genes to improve seed number is the relationship between ovule number and fertility, which respectively represent the quantity and quality of ovules. A few of these studies involving ovule number regulators also investigated ovule fertility, showing that most of these genes (including *MOB1A*, *HAP13*, *DET2*, *RGA2*, *GAI*, *ANT*, *miR164*, *EIF4A1*, *HEMN1*) affect them in the same direction, except for NERD1. This indicates that ovule number and fertility can be simultaneously improved. It should be noted that some of these genes (*LUG*, *NERD1*, *ONA2*, *PIN1*, *CKX3*, *CKX5*, *AHP6*, *RGL2*) also have pleiotropic effects on other characteristics/traits, such as flower number, plant height, and so on, in line with their expression activity in multiple organs. Therefore, special attention should be paid to their side effects when using these genes. It should also be noted that many of the collected genes affect ovule numbers in the different species (Supplementary Table S1), indicating their conserved functions in plants [64]. For example, *miR156*, *GAI*, *RGL1*, and *RGL2* can regulate ovule number in Arabidopsis, rice, and tomato [15,43,59].

Compared with other published reviews on ovule development and number, the current review collects the most complete ovule number regulators. More importantly, an integrated molecular network was constructed for the first time, which links the different types of regulators, including phytohormones, transcription factors, enzymes, other proteins, and micro-RNA. It represents a more comprehensive and in-depth cognition toward full understanding of ovule number regulation in plants. This knowledge is not only useful forbotany, but also for crop sciences in general. The constructed molecular network of ovule number regulation is still in the initial stages; since the number of known ovule number genes is restricted, only a small amount of information about the relationships between different genes inside and among pathways is available. The use of modern biotechnologies such as genome-wide association studies, genome editing, and bioinformatics will speed up the identification and verification of ovule number genes, paving the way for faster crop improvement. 

## Figures and Tables

**Table 1:** The list of key genes of ovule number in plants.

Species	Gene Name	Gene Model	Biological Function	References
Auxin (IAA) Signalling Pathway				
*Arabidopsis*	*PIN1*	*AT1G73590*	Component of the auxin efflux carrier	[1,3,32]
*Arabidopsis*	*SEUSS*	*AT1G43850*	Transcription co-regulator	[3,7,33]
*Arabidopsis*	*ETT/ARF3*	*AT2G33860*	Auxin response factors	[18,34,35]
*Arabidopsis*	*ARF6*	*AT1G30330*	Auxin response factors	[18]
*Arabidopsis*	*ARF8*	*AT5G37020*	Auxin response factors	[18]
*Arabidopsis*	*MOB1A*	*AT5G45550*	Promotes auxin signalling	[35,36]
*Arabidopsis*	*HAP13*	*AT1G60780*	Multiple post-Golgi trafficking pathways	[36,37,38]
*Arabidopsis*	*ARF5*	*AT1G19850*	Act as a transcriptional activator	[3,39]
*Arabidopsis*	*YUC1*	*AT4G32540*	Auxin biosynthesis	[31,40]
*Arabidopsis*	*YUC4*	*AT5G11320*	Auxin biosynthesis	[31,40]
*Arabidopsis*	*REV*	*AT5G60690*	homeodomain-leucine zipper family	[31,41]
Cytokinin (CTK) signalling pathway				
*Arabidopsis*	*AHK2*	*AT5G35750*	Cytokinin oxidase/ dehydrogenase	[3,22,42]
*Arabidopsis*	*AHK3*	*AT1G27320*	cytokinin oxidase/dehydrogenase	[3,22,42]
*Arabidopsis*	*CRE*	*AT2G01830*	cytokinin oxidase/dehydrogenase	[3,22,42]
*Arabidopsis*, *Rice*	*CUC1*	*AT3G15170*	SAM formation during embryogenesis	[1,31,43,44,45]
*Arabidopsis*, *Rice*	*CUC2*	*AT5G53950*	SAM formation during embryogenesis	[1,31,43,44]
*Arabidopsis*, *Rice*	*CKX3*	*AT5G56970*	Catalyzes the degradation of CK	[31,42,46,47]
*Arabidopsis*, *Rice*	*CKX5*	*AT1G75450*	Catalyzes the degradation of CK	[31,42,46,47]
*Arabidopsis*	*CKX6*	*AT3G63440*	Catalyzes the oxidation of CK	[42]
*Arabidopsis*, *Rice*	*AHP6*	*AT1G80100*	CK sensor histidine kinases	[46,47]
*Arabidopsis*	*ARR1*	*AT3G16857*	Type-B Arabidopsis response regulator	[31,48]
*Arabidopsis*	*ARR10*	*AT4G31920*	Type-B Arabidopsis response regulator	[31,48]
*Arabidopsis*	*ARR12*	*AT2G25180*	Type-B Arabidopsis response regulator	[31,48]
*Arabidopsis*	*CRF2*	*AT4G23750*	Transcriptional activator, binds GCC-box	[14,27,31,49]
*Arabidopsis*	*CRF3*	*AT5G53290*	Transcriptional activator, binds GCC-box	[14,27,31,49]
*Arabidopsis*	*CRF6*	*AT3G61630*	Transcriptional activator, binds GCC-box	[14,27,31,49]
*Arabidopsis*	*UGT85A3*	*AT1G22380*	O-glucosylation of trans-zeatin and dihydrozeatin	[1,31]
*Arabidopsis*	*UGT73C1*	*AT2G36750*	O-glucosylation of trans-zeatin and dihydrozeatin	[1,31]
Brassinosteroids (BRs) signalling pathway				
*Arabidopsis*	*BIN2*	*AT4G18710*	Negative regulator in BR signal transduction pathway	[3,31,50]
*Arabidopsis*	*HLL*	*AT1G17560*	Binds to 23S rRNA in the mitochondrion	[14,31,50,51]
*Arabidopsis*	*BRI1*	*AT4G39400*	Specificity kinase activity acting on threonine/tyrosine	[3,31,50]
*Arabidopsis*, *Rice*	*BZR1*	*AT1G75080*	Transcriptional repressor binds to BR response element	[3,22,31,50]
*Arabidopsis*	*DET2*	*AT2G38050*	BR biosynthesis of the plant steroid	[3,27,31,50]
*Arabidopsis*	*CYP85A1*	*AT5G38970*	Cytochrome p450 enzyme	[14]
*Arabidopsis*	*CYP85A2*	*AT3G30180*	Cytochrome p450 enzyme	[31,41]
*Arabidopsis*	*UNE16*	*AT4G13640*	Binds to phosphate starvation-regulated promoters	[27,31]
Gibberellins (GA) signalling pathway				
*Arabidopsis*, *Rapeseed*, *Tomato*, *Rice*	*RGA1*	*AT2G01570*	Repressor of the GA signalling pathway	[27,52,53]
*Arabidopsis*, *Rice*	*GID1A*	*AT3G05120*	Soluble gibberellin (GA) receptor	[27,52,54]
*Arabidopsis*, *Rice*	*GID1B*	*AT3G63010*	Soluble gibberellin (GA) receptor	[27,52,54]
*Arabidopsis*, *Tomato*, *Rice*	*GAI*	*AT1G14920*	Repressor of the GA signalling pathway	[27,31,52,54]
*Arabidopsis*, *Tomato*, *Rice*	*RGL2*	*AT3G03450*	Repressor of the GA signalling pathway	[27,52,55]
*Arabidopsis*	*REM22*	*AT3G46770*	DELLA interactor protein that mediates GA-regulate	[27,56]
*Arabidopsis*	*LNG4*	*AT1G18620*	Regulation of monopolar cell growth	[27,57]
*Arabidopsis*, *Rice*	*GA20OX3 *	*AT5G07200*	gibberellin biosynthetic process	[14,27,52,58]
*Arabidopsis*, *Tomato*	*GA3OX1*	*AT1G15550*	gibberellin biosynthetic process	[14,26,59]
*Tomato*	*SlGA20OX*	*Solyc03g006880*	gibberellin biosynthetic process	[14]
Other Signaling Pathways				
*Arabidopsis*	*AP2*	*AT4G36920*	Cadastral protein to repress C class floral homeotic gene	[14,31,57,60]
*Arabidopsis*	*PAN*	*AT1G68640*	DNA-binding transcription factor activity	[31,33]
*Arabidopsis*	*SPT*	*AT4G36930*	bHLH transcription factor	[3,35,48,61]
*Arabidopsis*	*ANT*	*AT4G37750*	Transcription activator binds to DNA sequence 5’- -3’	[14,50,62,63,64]
*Arabidopsis*	*LUG*	*AT4G32551*	Gynoecium, ON carpel development	[3,7,65]
*Arabidopsis*	*BEL1*	*AT5G41410*	SPL for CK-induced PIN1 expression in ovules	[14,31,60]
*Arabidopsis*, *Brassica*	*AG*	*AT4G18960*	Transcription regulation by RNA polymerase II	[14,31,60]
*Arabidopsis*	*SHP1*	*AT3G58780*	Transcription regulation by RNA polymerase II	[66,67,68]
*Arabidopsis*	*SHP2*	*AT2G42830*	Transcription regulation by RNA polymerase II	[66,67,68]
*Arabidopsis*	*STK*	*AT2G01930*	Transcriptional regulator that binds to GA-rich elements	[31,57,66,68]
*Arabidopsis*	*SEP1*	*AT5G15800*	determine the identity of petals, stamens, and carpels	[66]
*Arabidopsis*	*SEP2*	*AT3G02310*	determine the identity of petals, stamens, and carpels	[66]
*Arabidopsis*	*SEP3*	*AT1G24260*	determine the identity of petals, stamens, and carpels	[66]
*Arabidopsis*	*CRC*	*AT1G69180*	TFs required for the initiation of nectary development	[5]
*Arabidopsis*	*INO*	*AT1G23420*	formation and abaxial-adaxial asymmetric growth of ovule outer integument	[50,64,69]
*Arabidopsis*, *Rice*	*EPFL2*	*AT4G37810*	EPFL9 ligand for ER family receptors and ERL1/2 pathway	[45,70]
*Arabidopsis*	*ERL1*	*AT5G62230*	Redundantly involved with ER in procambial development regulation	[45,69,70]
*Arabidopsis*	*ERL2*	*AT5G07180*	Redundantly involved with ER in procambial development regulation	[45,69]
*Arabidopsis*, *Tomato*	*miR156*	*AT3G05040*	Nucleocytoplasmic transporter	[16]
*Arabidopsis*	*miR164*	*AT2G47585*	Transcription activator of STM and KNAT6	[27,44]
*Arabidopsis*	*EIF4A1*	*AT3G13920*	Cap recognition bind mRNA to ribosome	[71]
*Arabidopsis*	*SAP*	*AT5G35770*	Cadastral protein to repress C class floral homeotic gene	[72]
*Arabidopsis*	*ER*	*AT2G26330*	Receptor kinase of ERL1/2, regulates aerial architecture	[45]
*Arabidopsis/Nicotiana attenuata*	*NERD1*	*AT2G16485*	DNA methylation on cytosine, gene silencing by RNA	[22,31]
*Arabidopsis*	*ONA2*	*AT3G60660*	Regulation of microtubule polymerization	[22,31]
*Arabidopsis*	*SIN2*	*AT2G41670*	GTPase that functions in mitochondrial ribosome	[3,31,51]
*Arabidopsis*	*ASHH2*	*AT1G77300*	BR induced gene expression and histone H3 trimethylation	[3,73]
*Arabidopsis*	*HEMN1*	*AT5G63290*	Tetra pyrrole biosynthesis	[37,74]

## Data Availability

Data are contained within the article and its supplementary file.

## Supplementary Materials

The following are available online at https://www.mdpi.com/article/10.3390/ijms222312965/s1.
